# The mate recognition protein gene mediates reproductive isolation and speciation in the *Brachionus plicatilis* cryptic species complex

**DOI:** 10.1186/1471-2148-12-134

**Published:** 2012-08-01

**Authors:** Kristin E Gribble, David B Mark Welch

**Affiliations:** 1 Marine Biological Laboratory, 7 MBL Street, Woods Hole, MA, 02543, USA

**Keywords:** Mate recognition, Reproductive isolation, Speciation, Concerted evolution, Gene family

## Abstract

**Background:**

Chemically mediated prezygotic barriers to reproduction likely play an important role in speciation. In facultatively sexual monogonont rotifers from the *Brachionus plicatilis* cryptic species complex, mate recognition of females by males is mediated by the Mate Recognition Protein (MRP), a globular glycoprotein on the surface of females, encoded by the *mmr-b* gene family. In this study, we sequenced *mmr-b* copies from 27 isolates representing 11 phylotypes of the *B. plicatilis* species complex, examined the mode of evolution and selection of *mmr-b*, and determined the relationship between *mmr-b* genetic distance and mate recognition among isolates.

**Results:**

Isolates of the *B. plicatilis* species complex have 1–4 copies of *mmr-b*, each composed of 2–9 nearly identical tandem repeats. The repeats within a gene copy are generally more similar than are gene copies among phylotypes, suggesting concerted evolution. Compared to housekeeping genes from the same isolates, *mmr-b* has accumulated only half as many synonymous differences but twice as many non-synonymous differences. Most of the amino acid differences between repeats appear to occur on the outer face of the protein, and these often result in changes in predicted patterns of phosphorylation. However, we found no evidence of positive selection driving these differences. Isolates with the most divergent copies were unable to mate with other isolates and rarely self-crossed. Overall the degree of mate recognition was significantly correlated with the genetic distance of *mmr-b.*

**Conclusions:**

Discrimination of compatible mates in the *B. plicatilis* species complex is determined by proteins encoded by closely related copies of a single gene, *mmr-b*. While concerted evolution of the tandem repeats in *mmr-b* may function to maintain identity, it can also lead to the rapid spread of a mutation through all copies in the genome and thus to reproductive isolation. The *mmr-b* gene is evolving rapidly, and novel alleles may be maintained and increase in frequency via asexual reproduction. Our analyses indicate that mate recognition, controlled by MMR-B, may drive reproductive isolation and allow saltational sympatric speciation within the *B. plicatilis* cryptic species complex, and that this process may be largely neutral.

## Background

Identifying the mechanisms of speciation is one of the central pursuits in evolutionary biology. Evidence is mounting that prezygotic reproductive isolation may occur more quickly than post-zygotic isolation, effectively preventing sympatric populations from interbreeding [1-5]. Pheromone-based mate recognition has become a focus in the study of prezygotic isolation due to the apparent high specificity in signaling, the vast diversity of signaling systems between species, its independence from environmental differences, and the direct effect of chemical cues in preventing matings between divergent types [6,7].

The evolution of chemically mediated prezygotic barriers to reproduction may play an important role in speciation. As the signal for mate recognition diverges within or between populations, formerly compatible strains may become reproductively isolated, initiating the formation of new species. While examples of compounds involved in mate choice are known in organisms ranging from yeast to humans, and the resulting mating behavior is well described, the genetic bases and evolution of these chemical cues are thoroughly characterized for relatively few species (e.g. [8-10]). Often, the correlation between cue diversity, evolution, and mating cannot be made directly due to the difficulty in characterizing the mating cue, identifying the gene(s) giving rise to the (often extremely complex) chemical signal, and conducting mate recognition studies in the same populations.

In this study, we employed molecular genetics, phylogenetics, and behavioral assays to examine the evolution and role of mate choice within the *Brachionus plicatilis* group, a cryptic species complex of monogonont rotifers. Monogonont rotifers are cyclically parthenogenetic; they generally reproduce asexually, but through a quorum sensing process or due to environmental factors such as temperature, food conditions, or pH, will produce males and undergo sexual reproduction [11-14]. Members of the *B. plicatilis* group are found in inland saline environments around the world, often as sympatric species. Molecular phylogenetics based on *coxI* and *its1* suggest the group is composed of 13 – 24 morphologically inconclusive phylotypes [15-18], although only four species have been formally described: *B. rotundiformis* tschugunoff 1921; *B. plicatilis* of müller 1786, *B. ibericus* ciros-perez 2001, and *B. manjavacas* fontaneto 2007 [19-21]. The species complex is split into two main clades, “A” and “B,” with *B. rotundiformis* in clade “C,” and additional phylotypes falling into additional groups [16,17]. Crosses between different phylotypes have shown a gradient in mate recognition and copulation between members of the species complex [17,22]*.*

While much of the empirical and theoretical literature about mate choice focuses on female selection of male traits, males appear to play the predominant role in selecting a mate among *B. plicatilis* populations. A male rotifer randomly encounters a female, circles the female closely, localizes on her corona, and—if the female is recognized as compatible—copulates by hypodermic insemination [23]. Recognition is mediated by the Mate Recognition Protein (MRP), a glycoprotein on the surface of females [24,25]. Removal of MRP by EDTA causes cessation of male circling, and re-application of MRP to the surface of conspecific females, to females of a reproductively isolated phylotype, or even to plastic beads, restores male circling and copulatory behavior [26].

The gene encoding MRP is the eponymous member of the MRP Motif Repeat (MMR) gene family [25,27]. MMR genes share the same basic structure: a signal peptide sequence, followed by one to nine nearly identical 276 bp (*mmr-a*) or 261 bp (*mmr-b*) “full” repeats, with a terminal repeat of 243 bp in which the final 11 (in *mmr-a*) or 6 (in *mmr-b*) codons are replaced by 4 or 2 non-homologous codons. MMR-B genes were recently shown through RNAi knockdown to be responsible for mate recognition in *B. manjavacas*[25,28].

Here we describe the diversity and evolution of *mmr-b* within 27 clonal isolates representing 11 phylotypes, or probable species, within the *B. plicatilis* species complex. Using results of assays of mating behavior between phylotypes, we correlated mate recognition with genetic distance to determine the relationship between *mmr-b* and prezygotic reproductive isolation. Insights into the genetic basis of mate recognition and the mode of evolution of *mmr-b* indicate that mate recognition, mediated by MMR-B, plays a driving role in speciation within the cryptic species complex.

## Results

**Figure 1:**
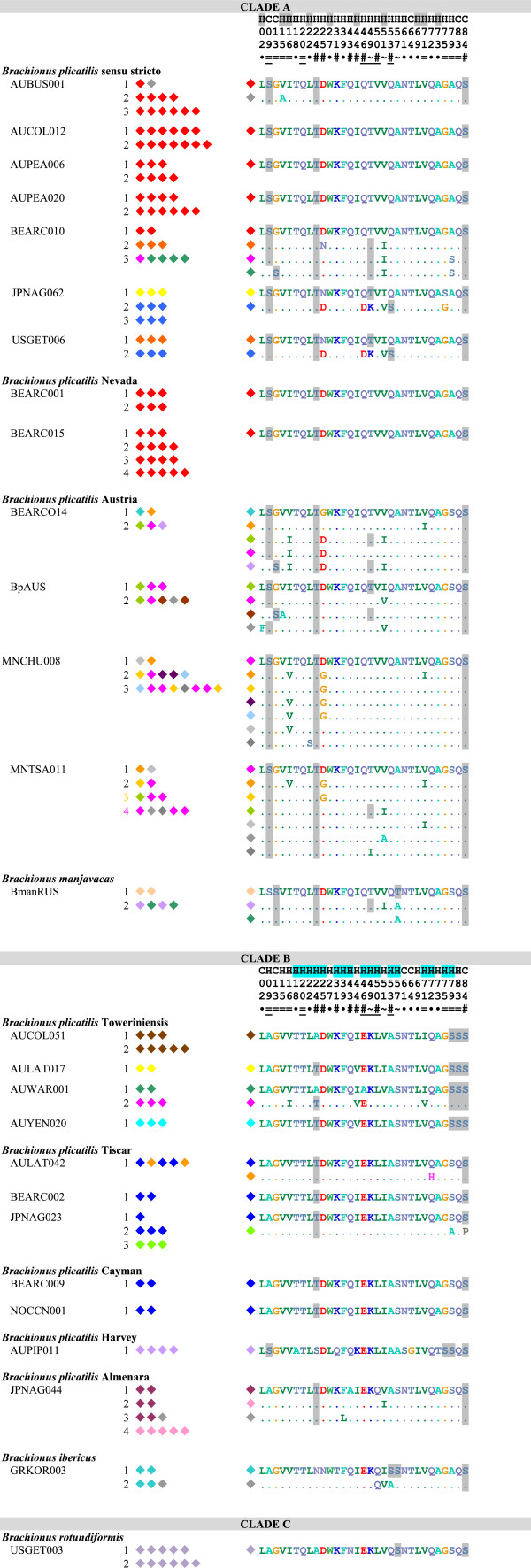
**Amino acid identity among full repeat motifs of MMR-B.** All gene copies are listed for each species. Each diamond represents one repeat unit in the gene copy; colors indicate identity of repeats. Grey represents repeats with unique amino acid sequences, all other colors represent repeats found multiple times within Clade A or Clade B. Numbered polymorphic positions are shown to the right, with differences from the first repeat of the shortest gene copy shown for each isolate; predicted phosphorylated amino acids are shaded. The consensus prediction of the position being part of a coil (C) or helix (H) is shown above the position numbers, with positions predicted to be buried in the hydrophobic core shaded. Symbols below the position numbers indicate if the polymorphism is unique to a single repeat (·), alternates between only two (=) or almost always two (~) residues, or is more polymorphic (#). Underlining indicates synapomorphies between Clades A and B.

**Figure 2:**
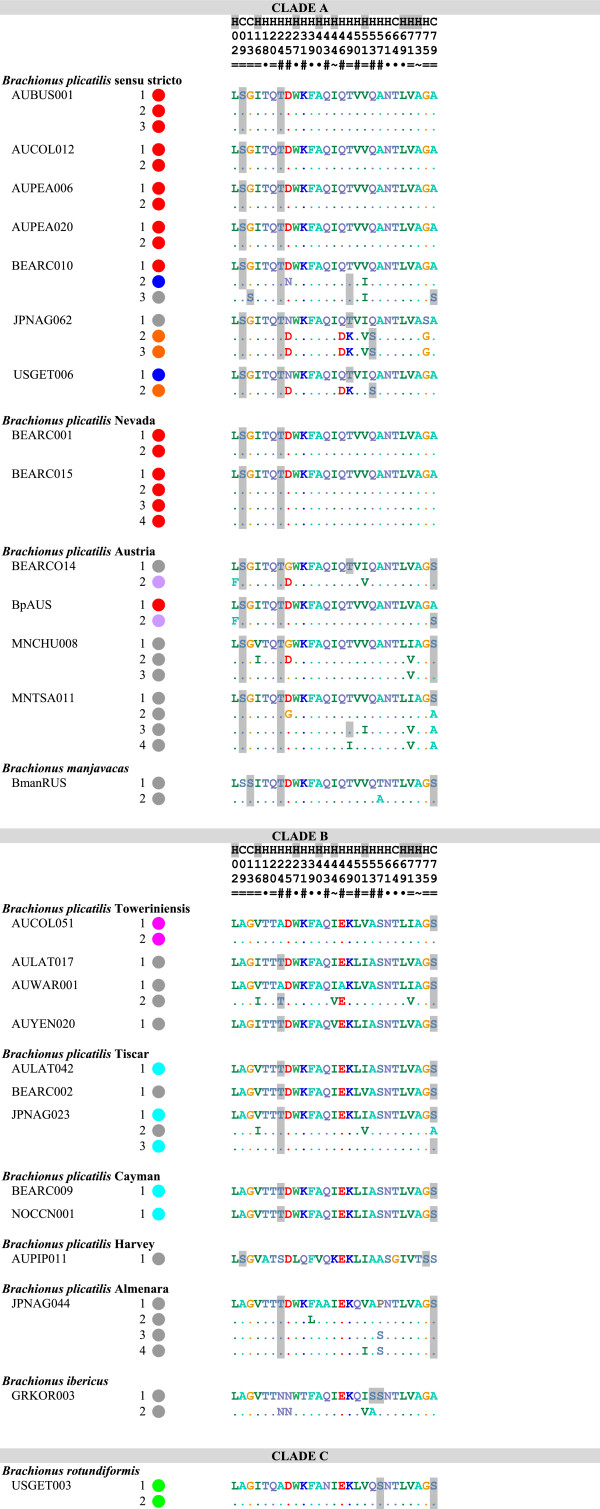
**Amino acid identity among terminal repeat motifs of MMR-B.** All gene copies are listed for each species. Each circle represents the number of repeat units in each gene copy; colors indicate identity of repeats. Grey represents repeats with unique amino acid sequences, all other colors represent repeats found multiple times within Clade A or Clade B. Numbered polymorphic positions are shown to the right, with differences from the first repeat of the shortest gene copy shown for each isolate; predicted phosphorylated amino acids are shaded. The consensus prediction of the position being part of a coil (C) or helix (H) is shown above the position numbers, with positions predicted to be buried in the hydrophobic core shaded. Symbols below the position numbers indicate if the polymorphism is unique to a single repeat (·), alternates between only two (=) or almost always two (~) residues, or is more polymorphic (#). Underlining indicates synapomorphies between Clades A and B.

We amplified, cloned, and sequenced *mmr-b* gene(s) from 27 isolates representing 11 phylotypes from Clades A, B, and C of the *B. plicatilis* cryptic species complex (Table 1). We found 1–4 copies of *mmr-b* in each isolate (Table 1, Figures 1 and 2); copies ranged in length from 554 bp (one full repeat and one terminal repeat) to 2382 bp (eight full repeats and one terminal repeat). The average number of copies and the average number of repeats were both significantly higher in Clade A (2.8 copies, 4.8 repeats) than B (1.7 copies, 3.9 repeats; two-tailed Mann–Whitney *U*-test, p < 0.05). Two copies of *mmr-b*, one in a Clade A *B. plicatilis* sensu stricto (s.s.) isolate and one in a Clade B Almenara isolate, contained single stop codons (at different positions) and were not included in phylogenetic analyses or analyses for selection. Both isolates had additional copies of *mmr-b*.

**Table 1:** **Isolates and *mmr-b *copiesb **

**Species or phylotype**	**Isolate**	***mmr-b *copy bases (repeats)**
CLADE A:		
*B. p.* sensu stricto	AUBUS001	817 (3)
	AUBUS001	1337 (5)
	AUBUS001	1860 (7)
	AUCOL012	1860 (7)
	AUCOL012	2121 (8)
	AUPEA006	1077 (4)
	AUPEA006	1338 (5)
	AUPEA020	1338 (5)
	AUPEA020	1862 (7)
	BEARCO10	816 (3)
	BEARCO10	1077 (4)
	BEARCO10	1587 (6)
	JPNAG062	1076 (4) A
	JPNAG062	1076 (4) B
	JPNAG062	1078 (4)
	USGET006	1076 (4) A
	USGET006	1076 (4) B
*B.* sp. Austria	BEARCO14	816 (3)
	BEARCO14	1077 (4)
	BpAUS	1077 (4)
	BpAUS	1598 (6)
	MNCHU008	816 (3)
	MNCHU008	1599 (6)
	MNCHU008	2382 (9)
	MNTSA011	816 (3) A
	MNTSA011	817 (3) B
	MNTSA011	1077 (4)
	MNTSA011	1599 (6)
*B.* sp. Nevada	BEARCO01	1077 (4)
	BEARCO01	1338 (5)
	BEARCO15	1077 (4)
	BEARCO15	1338 (5) A
	BEARCO15	1338 (5) B
	BEARCO15	1599 (6)
*B. manjavacas*	BmanRUS	816 (3)
	BmanRUS	1338 (5)
CLADE B:		
*B.* sp. Towerinniensis	AUCOL051	1077 (4)
	AUCOL051	1599 (6)
	AULAT017	817 (3)
	AUWAR011	817 (3)
	AUWAR011	1077 (4)
	AUYEN020	1077 (4)
*B.* sp. Tiscar	AULAT042	1599 (6)
	BEARC002	817 (3)
	JPNAG023	554 (2)
	JPNAG023	1076 (4) A
	JPNAG023	1076 (4) B
*B.* sp. Cayman	BEARCO09	816 (3)
	NOCCN001	816 (3)
*B.* sp. Harvey	AUPIP011	1338 (5)
*B.* sp. Almenara	JPNAG044	804 (3) A
	JPNAG044	816 (3) B
	JPNAG044	1077 (4)
	JPNAG044	1599 (6)
*B. ibericus*	GRKOR003	816 (3)
	GRKOR003	1077 (4)
CLADE C:		
*B. rotundiformis*	USGET003	1605 (6)
	USGET003	1867 (7)

The translated amino acid sequences of repeats are unique to a single phylotype with four exceptions (Figures 1 and 2): All full and terminal repeats in all Clade A Nevada isolates are identical to the most common sequence found in *B. plicatilis* s.s. isolates; one terminal repeat of an Austria isolate is identical to the predominant terminal repeat in *B. plicatilits* s.s.; all full and terminal Clade B Cayman repeats are identical to the most common repeat in Clade B Tiscar; and one copy of *mmr-b* in the *B. plicatilis* s.s. isolate BEARCO10 is made up of full repeats found in Clade A Austria and *B. manjavacas* (colored magenta and dark green, respectively, in Figure 1).

Within many phylotypes, the full repeats of a gene copy generally encode identical amino acid sequences. Of note are the sequences shared by the *B. plicatilis* s.s. isolates BEARCO10, JPNAG062, and USGET006 colored blue, yellow, and orange in Figure 1, which each differ by 2–3 amino acids from the common *B. plicatilis* s.s. sequence (colored red in Figure 1). Terminal and full repeats from the Clade A Austria phylotype are remarkable in encoding a wide variety of amino acid sequence types within and between gene copies in an isolate; many of these types appear in different positions in different isolates.

### Structural and post-translational polymorphisms in *mmr-b*

The peptide encoded by each *mmr-b* repeat is predicted to form a series of alpha helices, with each helix composed of a hydrophobic side dominated by aliphatic residues that would be buried within the globular protein, and a polar side exposed to the extracellular environment [25,27]. Different secondary structure prediction programs vary in the confidence with which they predict the central region (residues 15–60) of different repeat copies to form 1, 2, or 3 helices, but for all copies this region can be represented as a single helix with a hydrophobic side and negatively charged polar side. The hydrophobic portion of each helix is largely invariant across repeat copies, with most changes being highly conserved. The exceptions are the repeats found in the Harvey isolate, with a positively charged K at position 44 while all other repeats in all other isolates have an aliphatic I or V, and a polar T at position 73 while all other repeats but one have an aliphatic A. Most repeats in the JPNAG062/USGET006 group contain a T to K change and two changes from uncharged amino acids to D, resulting in a more highly charge surface.

Potential phosphorylation sites in repeats differ substantially between clades (Figures 1 and 2). Only the predicted phosphorylation of S84, in the loop region connecting one repeat to the next, is conserved through the entire species complex (though it has been lost in one repeat in Clade B Tiscar). Predicted phosphorylation of T24 is relatively well conserved between Clades A and B in full and terminal repeats, with scattered losses in Clade B. Most Clade A repeats are potentially phosphorylated at S9 on the exposed side of the first helix, though phosphorylation is shifted to S13 in some repeats, including all of those in *B. manjavacas*. Residues 50 and 53 are variably potentially phosphorylated throughout Clade A; unlike all other *B. plicatilis* s.s. repeats, those in the JPNAG062/USEGET006 group are potentially phosphorylation at these positions. Repeats from Clade B have fewer potential phosphorylation positions, with none synaptomorphic for the clade. Full repeats from Clade B Harvey and Towerinniensis have more potential phosphorylation sites, with all Harvey repeats containing S9 unique to Clade B, and all Towerinniensis repeats having a unique phosphorylation motif around S79 and having a unique S83. The glucosaminoglycan motif QSGK at residues 83–86 was conserved in nearly all repeats. In all repeats from the Clade B Towerinniensis, the motif was altered to SSGK, and the motif was lost in one repeat from Clade B Tiscar.

### MRP gene trees

Because the lack of obvious orthology between full repeats made aligning complete gene copies ambiguous, we used the repeat rather than the gene as the unit for analysis. We analyzed the terminal repeat separately from the set of full-length non-terminal repeats, with repeats of two *B. manjavacas mmr-a* copies as outgroups [25,27].

**Figure 3:**
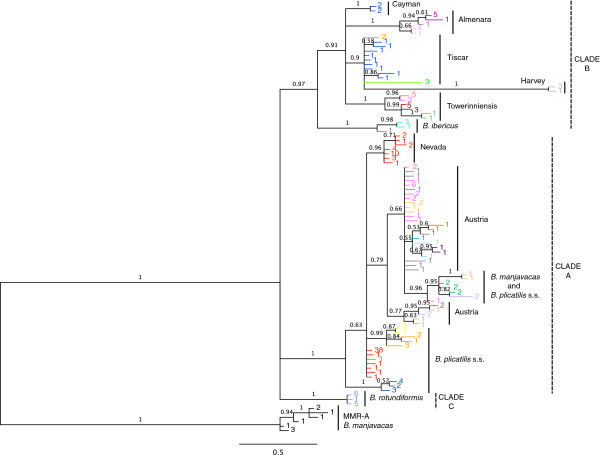
**Bayesian trees of *mmr-b *full repeat coding sequences.** Posterior probabilities are shown along the branches. Numbers at the tip of each branch indicate the number of identical repeats represented by that branch; colors are the same as in Figure 1. Phylotype and major clade designations are shown to the right.

**Figure 4:**
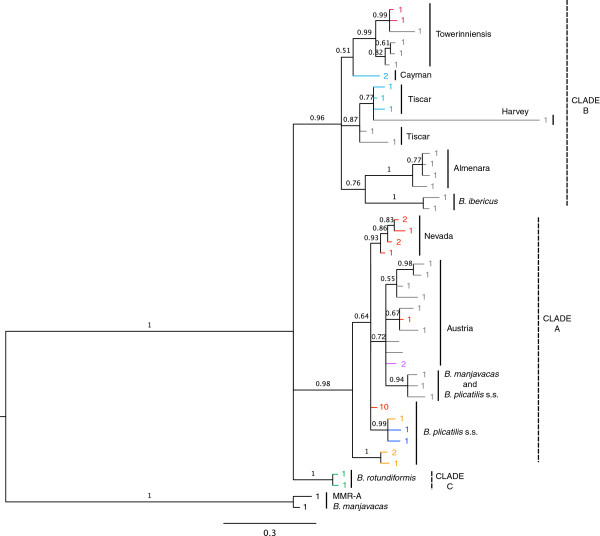
**Bayesian trees of *mmr-b *terminal repeat coding sequences.** Posterior probabilities are shown along the branches. Numbers at the tip of each branch indicate the number of identical repeats represented by that branch; colors are the same as in Figure 2. Phylotype and major clade designations are shown to the right.

**Figure 5:**
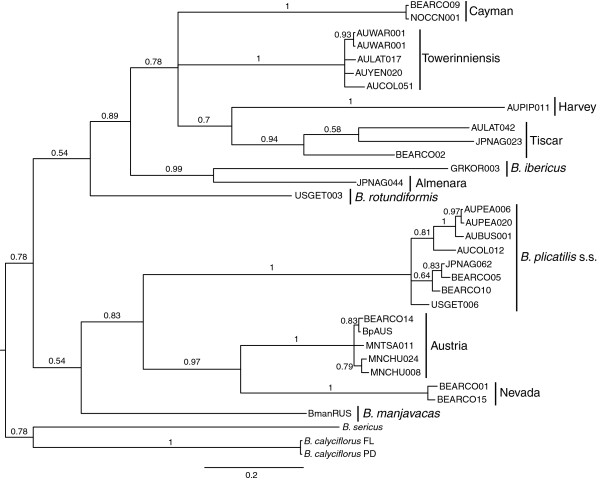
**Bayesian trees of *coxI *coding sequences.** Posterior probabilities are shown along the branches. Names at the tip of each branch designate isolates. Phylotype and major clade designations are shown to the right.

**Figure 6:**
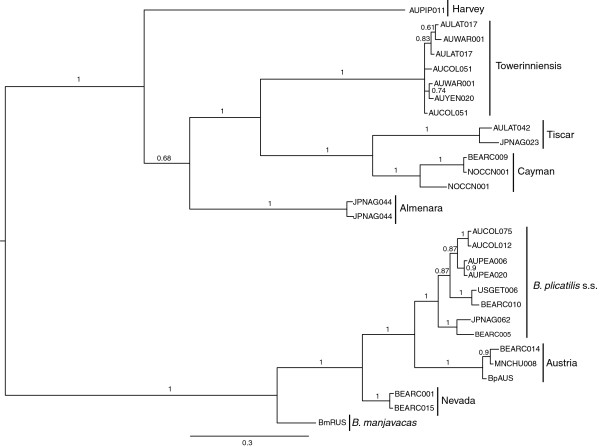
**Bayesian trees of *hsp82 *coding sequences.** Posterior probabilities are shown along the branches. Names at the tip of each branch designate isolates. Phylotype and major clade designations are shown to the right.

The gene trees of both full and terminal *mmr-b* repeats (Figures 3 and 4) recapitulated the phylogeny of the *B. plicatilis* species complex inferred from *coxI* and *hsp82* (Figures 5 and 6), and from *its*[15-17]. Both full and terminal repeats fell into two main clades, one containing isolates of *coxI*-defined Clade A phylotypes (*B. plicatilis* s.s., Nevada, Austria, and *B. manjavacas*) and another containing *coxI*-defined Clade B phylotypes (*B. ibericus,* Almenara, Tiscar, Harvey, Cayman and Towerinniensis). A third, Clade C, contained *B. rotundiformis*. Although most isolates had more than one copy of *mmr-b*, there was little evidence of shared inheritance of nucleotide polymorphisms between phylotypes.

The full repeats generally clustered by phylotype and not by gene copy, position within a gene copy, or geographic origin of the isolate. Repeats within Clade B were monophyletic for each phylotype, except for those of Tiscar, which included a long branch to the Harvey phylotype, a possible artifact caused by rapid or poorly-modeled evolution of the Harvey phylotype. The fact that all repeats from the Cayman phylotype were identical with most repeats from the Tiscar phylotype at the amino acid level was not apparent in the nucleotide phylogeny, suggesting strong purifying selection on a common amino acid sequence. Similarly, despite the amino acid identity of most Clade A Nevada repeats with most *B. plicatilis* s.s. repeats, there was strong support for the monophyly of the Nevada phylotype at the nucleotide level. Other Clade A phylotypes were largely unresolved: A poorly supported clade of repeats from the Austria phylotype included a well-supported clade of *B. plicatilis* s.s. and *B. manjavacas* repeats, and other *B. plicatilis* s.s. repeats appeared across Clade A, including a well-supported basal clade of repeats from the JPNAG062 and USGET006 isolates (colored blue in Figures 1 and 2). One repeat within the pseudogene of a *B. plicatilis* s.s. isolate (BEARC010) was identical to the repeats within one copy of *B. manjavacas*, suggesting recent introgression between these two species. Similar to the full repeats, copies of the terminal repeats clustered within phylotypes, with the exception of the poly- and paraphyletic *B. plicatilis* s.s. and *B. plicatilis* Austria complex. Analyses of codon third positions of the alignments provided the same basic tree topologies for full and terminal repeats, though with lower support and additional polytomies.

### Mode of evolution and selection

**Figure 7:**
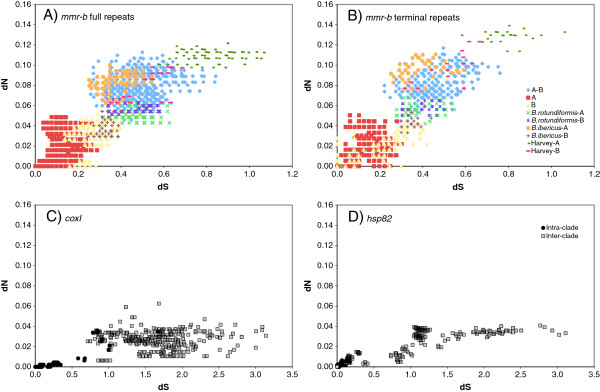
**dN vs dS for coding sequences.** (**A**) *mmr-b* full repeats; (**B**) *mmr-b* terminal repeats; (**C**) *coxI*; (**D**) *hsp82*. Differently colored symbols indicate different intra- and inter-clade or clade-phylotype pairwise comparisons.

The majority of codons in all repeats are under strong purifying selection. Pairwise comparisons of non-synonymous (dN) and synonymous (dS) accumulation between full repeats and between terminal repeats (Figure 7) show that dN/dS was generally in the range of 0.10 – 0.26, with a significantly higher average rate in terminal repeats (0.144 for full repeats v. 0.157 for terminal repeats, p < 0.001, two-tailed Mann–Whitney *U*-test). Excluding *B. plicatilis* Harvey, mean intra-clade nonsynonymous and synonymous variation between repeats were both significantly higher in Clade B (0.03088 and 0.2535, respectively) than Clade A (0.01223 and 0.1249, respectively; two-tailed Mann–Whitney *U*-test, p < 0.0001). Consistent with the unresolved position of *B. rotundiformis*, its repeats were similarly divergent from Clade A and Clade B repeats at both nonsynonymous and synonymous positions. Repeats from *B. plicatilis* Harvey, which together have a very long branch in the trees in Figures 3 and 4, have a significantly higher dN (0.08314) and dS (0.5391) than other Clade B repeats (two-tailed Mann–Whitney *U*-test, p < 0.0001).

Both dN and dS of *mmr-b* differed from those of housekeeping genes *hsp82* and *coxI* for the same isolates (Figure 7 and Table 2). For *hsp82* and *coxI*, dN remained low for both within species and between species comparisons, reaching a plateau around 0.04, while dS rose rapidly, with many interspecies differences exceeding 1.0. In contrast, dN rose steadily with dS for *mmr-b* full and terminal repeats; dN was more than twice and dS less than half the value in housekeeping genes. The mean dN/dS of all pairwise comparisons of full or terminal repeats for *mmr-b* (0.144 and 0.157, respectively) was significantly higher than those for *hsp82* (0.031) or *coxI* (0.021; p < 0.001, two-tailed Mann–Whitney *U*-test).

**Table 2:** Rates of change in coding sequences

	**dN**	**dS**	**dN/dS**
*coxI*	0.028 (0.015)	1.440 (0.638)	0.021 (0.014)
*hsp82*	0.022 (0.014)	0.951 (0.749)	0.031 (0.032)
*mmr-b* main repeats	0.044 (0.035) *	0.287 (0.204) *	0.144 (0.088) *
*mmr-b* final repeats	0.050 (0.036) * ^**+**^	0.303 (0.183) * ^**+**^	0.157 (0.095) * ^**+**^

Mean of all pairwise comparisons within the B. plicatilis species complex, with (s.d.); * indicates a value statistically significantly different than that for *hsp82* or *coxI*; + indicates a value statistically significantly higher than that for *mmr-b* main repeats (Mann–Whitney *U*-test, p < 0.001, two tailed test).

**Figure 8:**
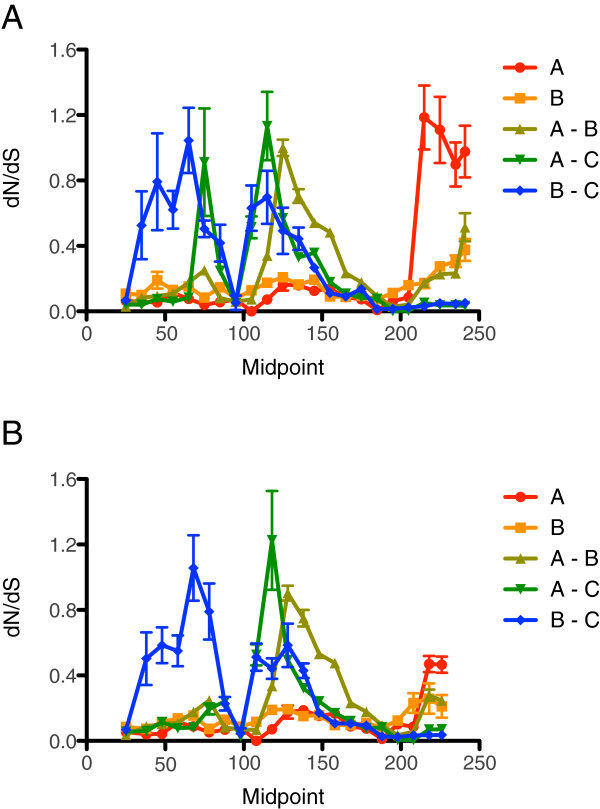
**Mean pairwise dN/dS values along a sliding window of the *mmr-b *repeats.** (**A**) full repeats; (**B**) terminal repeats. Error bars indicate standard error.

There is considerable variation in dN, dS, and dN/dS along the length of the *mmr-b* repeats. Around codons 45–50, pairwise comparisons between Clade A and Clade B repeats showed an elevated dN/dS of 0.8-1.3 (Figure 8); comparisons of the diverging isolates of *B. plicatilis* s.s. (BEARC010, JPNAG062, and USGET006) with other Clade A repeats showed a similar peak (not shown). In addition, comparisons of the divergent Harvey isolate with other Clade B repeats showed a region of elevated dN/dS around codons 60–70. Many comparisons showed a highly elevated dN/dS at the end of the full repeats, due to isolated nonsynonymous differences in the absence of synonymous differences.

To determine if elevated dN/dS in specific regions of particular lineages is driven by positive selection, we conducted site (Table 3) and branch-site (Table 4) tests for positive selection using codeml. Due to strong codon usage bias in *mmr-b*, we estimated codon frequency using observed frequencies (codon frequencies = 3); other methods gave similar results but generally estimated a higher transition:transversion ratio. An evolutionary model allowing three possible values of dN/dS over the whole tree of full repeats estimated that >95% of sites are under strong purifying selection (dN/dS < 0.2) while selection is more relaxed at the remaining sites (dN/dS ~ 0.6). This model was significantly better than one with a single dN/dS estimate of 0.08 (*i.e.* the M0 vs. M3 test for variation in selective pressure; Table 3; *χ*^2^ test, p < 0.0001). Models that allowed a proportion of codons across all branches of the tree to have a dN/dS > 1 were not significantly better than those that did not allow dN/dS to exceed one (*i.e.* the M2 vs. M1 and M8 vs. M7 tests for positive selection; results were similar for terminal repeats). Models allowing the major branches of Clade A, or the major branches of Clade B, or either or both of the branches leading to the JPNAG062/USGET006 repeats to have a different distribution of dN/dS from the rest of the tree (*i.e.* M2 or M3 with model = 2) did not estimate that the branch(es) tested had any proportion of codons with dN/dS > 1. Tests of branches leading to other isolates (Tiscar, Almenara, *etc.*) yielded similar results, with two exceptions: the branch leading to Clade B Harvey, estimated to have ~21% of codon positions with dN/dS ~1.5, and the branch leading to *B. ibericus*, estimated to have ~6% of codons positions with dN/dS ~2.5 (Table 4). In neither case was the model significantly better than a model in which the dN/dS of the branch was fixed at 1 (the branch site test of selection [29]) In both cases, however, the model allowing the branch to have a distribution of dN/dS different from the rest of the tree was significantly better than one in which all branches had the same dN/dS distribution [30], suggesting that the branches are under relaxed selection. We also performed this test on the branch leading to Clade B, which had a mildly elevated dN/dS (~0.7), but the difference between the models was not significant.

**Table 3:** Site models and tests of selection

**Model**	**Parameter estimates**	**ĸ**	**lnL**	**p**
M0	ω = 0.070	1.01	−1961.39	**<<0.001**
M3	p_0_ = 0.585 p_1_ = 0.383, p_2_ = 0.032	0.98	−1922.15	
	ω_0_ = 0.001, ω_1_ = 0.151, ω_2_ = 0.615			
M1	p_0_ = 0.952, p_1_ = 0.048,	1.13	−1946.80	--
	ω_0_ = 0.057 ω_1_ = 1.000			
M2	p_0_ = 0.952, p_1_ = 0.048, p_2_ = 0.000,	1.13	−1946.80	
	ω_0_ = 0.057, ω_1_ = 1.000, ω_2_ = 42.1			
M7	p = 0.260, q = 2.948	0.94	−1924.53	--
M8	p_0_ = 1.000, p = 0.260	0.94	−1924.53	
	q = 2.95, (p_1_ = 0.000), ω = 2.51			

Model refers to the Nsites value in codeml (e.g. M0 is nSites = 0); other parameter values for all tests were: codon frequency =3, model =0, omega = 0; ω is dN/dS and p_n_ is the proportion of codons with ration ω_n_; κ is the transition:transversion ratio; LnL is the log likelihood, p is the result of *χ*^2^ test, which was not performed when the likelihoods were identical.

**Table 4:** Branch-Site models and tests of selection

**Branch**	**model**	**Parameter estimates**					**lnL**	**p**
		**Site class**	**0**	**1**	**2a**	**2b**		
Harvey	M2 H_0_	p_n_	0.712	0.044	0.230	0.014	−1939.35	0.65
		back ω	0.046	1.000	0.046	1.000		
		fore ω	0.046	1.000	1.000	1.000		
	M2 H_1_	p_n_	0.744	0.046	0.197	0.012	−1939.25	
		back ω	0.047	1.000	0.047	1.000		
		fore ω	0.047	1.000	**1.440**	**1.440**		
Ibericus	M2 H_0_	p_n_	0.866	0.040	0.090	0.004	−1945.76	0.29
		back ω	0.055	1.000	0.055	1.000		
		fore ω	0.055	1.000	1.000	1.000		
	M2 H_1_	p_n_	0.897	0.042	0.058	0.003	−1945.51	
		back ω	0.055	1.000	0.055	1.000		
		fore ω	0.055	1.000	**2.437**	**2.437**		
Harvey	M3 H_1_	p_n_	0.587	0.249	0.115	0.049	−1916.07	**0.0005**
		back ω	0.003	0.221	0.004	0.221		
		fore ω	0.003	0.221	1.497	1.497		
Ibericus	M3 H_1_	p_n_	0.622	0.357	0.013	0.008	−1925.01	**0.017**
		back ω	0.004	0.198	0.004	0.198		
		fore ω	0.004	0.198	**2.549**	**2.549**		
Clade B	M3 H_1_	p_n_	0.605	0.356	0.025	0.015	−1921.68	0.33
		back ω	0.002	0.169	0.002	0.169		
		fore ω	0.002	0.169	0.694	0.694		

Branch refers to the branch allowed to have a dN/dS distribution independent of the rest of the tree; Model refers to the Nsites value in codeml; M2 is the branch-site test of positive selection; M3 is the test of divergent selection pressure. For M2:H_0_ the foreground ω (branch tested) is fixed at 1; (omega = 1; fix_omega = 1); for M2:H_1_ ω is estimated from the data (omega = 0); for M3:H_0_ all branches have the same distribution of ω (see Table 4, “M3” for values); for M3:H_1_ the branch values are estimated independently from the data. Other parameter values were codon frequency =3, and for M3, ncat = 3. dN/dS and p_n_ is the proportion of codons with ratio ω_n_; κ is the transition:transversion ratio; LnL is the log likelihood, p is the result of *χ*^2^ test; bold indicates test that were statistically significant.

### Mate recognition of *Brachionus* isolates

We conducted a series of targeted reciprocal mating assays within and between isolates. Rates of copulation were always highest in self-crosses; with the exception of some crosses within and between *B. plicatilis* s.s. and Clade A Austria, rates of circling and copulation were always highest within phylotypes (Table 5). Within *B. plicatilis* s.s., males from an isolate with the dominant form of *B. plicatilis* s.s. MMR-B repeat (AUBUS001) had significantly lower copulation rates with females from the isolate JPNAG062 than with other *B. plicatilis* s.s. isolates, and males from JPNAG062 showed a greater preference for circling and copulating with AUBUS001 females than females of their own strain. AUBUS001 males had significantly higher rates of circling females from an Austria isolate than for a closely related *B. plicatilis* s.s. isolate, and similar rates of copulation. In crosses between Clades A and B, circling occurred at reduced rates. Copulation did occur between males of *B. plicatilis* s.s. and Clade B females, but at a significantly reduced rate than for self-crosses, and males of Clade A *B. manjavacas* did not copulate with Clade B females. Males of Clade B did not copulate with either of two Clade A phylotypes or outcross with other Clade B phylotypes.

**Table 5:** Circling and copulation in mating bioassays

**Males**		**Females**		**Circling (%)**	**SE**	**Sig**	**Copulation (%)**	**SE**	**Sig**
*B. plicatilis* s.s.(1)	AUPEA006	*B. plicatilis* s.s (1)	AUPEA006	11.6	1.2		48.1	12.5	
		*B. plicatilis* s.s (1)	AUBUS001	17.6	2.9	*	32.0	4.0	
		*B. manjavacas*	BmRUS	14.5	2.9		14.4	6.0	
		Towerinniensis	AUYEN020	9.6	2.1		14.5	6.2	*
*B. plicatilis* s.s (1)	AUBUS001	*B. plicatilis* s.s (1)	AUBUS001	24.4	2.3		47.4	4.9	
		*B. plicatilis* s.s (2)	JPNAG062	28.3	3.0		8.3	3.0	***
		*B. plicatilis* s.s (1)	AUPEA006	8.4	2.0	**	35.4	9.7	
		Austria	MNCHU008	21.1	1.1		33.9	7.8	
		Tiscar/Japan	JPNAG023	3.8	1.5	**	3.7	3.7	*
*B. plicatilis* s.s (2)	JPNAG062	*B. plicatilis* s.s (2)	JPNAG062	6.4	1.7		12.2	7.9	
		*B. plicatilis* s.s (1)	AUBUS001	11.0	3.0		21.3	10.0	
*B. manjavacas*	BmRUS	*B. manjavacas*	BmRUS	11.4	1.8		34.0	6.0	
		*B. plicatilis* s.s. (2)	BEARCO10	9.8	1.7		4.2	2.8	
		*B. plicatilis* s.s (1)	AUPEA006	6.0	1.2		24.9	10.9	
		Towerinniensis	AUYEN020	4.4	1.2	*	0.0	0.0	***
Towerinniensis	AUYEN020	Towerinniensis	AUYEN020	12.8	1.7		34.5	5.7	
		*B. plicatilis* s.s (1)	AUPEA006	0.6	0.3	***	0.0	0.0	***
		*B. manjavacas*	BmanRUS	4.8	1.3	*	0.0	0.0	***
		Harvey	AUPIP011	0.0	0.0	**	0.0	0.0	**
Harvey	AUPIP011	Harvey	AUPIP011	0.2	0.2		12.5	12.5	
		Towerinniensis	AUYEN020	0.0	0.0		0.0	0.0	

Percentages shown for self-crosses are averages from multiple experiments. Parentheses following *B. plicatilis* s.s. indicate whether the isolate contains *mmr-b* sequence type 1 or type 2. Significance of differences between self- and out-crosses was calculated using data from single self-cross experiment conducted simultaneously with out-cross, which is different than the average from the multiple experiments shown. Significance determined using Mann-Whitney U-test (* for p < 0.05, ** for p < 0.005, *** for p < 0.0005, two-tailed test).

**Figure 9:**
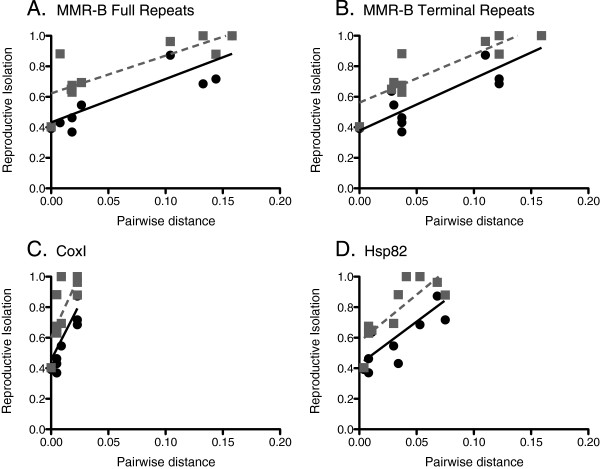
**Correlations between reproductive isolation and genetic distance.** (**A**) *mmr-b* full repeats; (**B**) *mmr-b* terminal repeats; (**C**) *coxI*; and (**D**) *hsp82*. Black circles represent reproductive isolation calculated from percent circling; grey squares indicate reproductive isolation calculated from percent copulation.

Genetic distance of MMR-B between pairs of isolates within the species complex was a good predictor of prezygotic reproductive isolation (Figure 9). The RI index, in which a value of 0 indicates 100% circling or copulation in outcrosses and a value of 1 indicates a complete lack of circling or copulation, was significantly positively correlated with amino acid pairwise distance for the full and terminal repeats of MMR-B (Pearson’s product–moment correlation coefficient, p < 0.01). Amino acid pairwise distances of the mitochondrial COXI and nuclear HSP82*,* were not significantly correlated with prezygotic reproductive isolation (p > 0.01, Pearson’s product–moment correlation coefficient), except for a positive correlation between Hsp82 pairwise distance and copulation RI (Pearson’s product–moment correlation coefficient, p < 0.01).

## Discussion

Exactly what role prezygotic isolation plays in speciation is an intensely debated and active area of research in evolutionary biology. As the genetic bases and evolutionary mechanisms of chemical mate recognition systems are well elucidated for only a handful of systems, additional evidence from a wider range of organisms is needed to determine whether and how changes in mate choice leading to reproductive isolation have a causative role in speciation. In this study, we examined the gene encoding the Mate Recognition Protein, *mmr-b*, within the *B. plicatilis* cryptic species complex. We sequenced *mmr-b* copies from 27 isolates representing 11 phylotypes, examined the evolution of *mmr-b* in the complex, and determined the relationship between *mmr-b* genetic distance and mate recognition among isolates from the complex.

### Evolution of mmr-b within the cryptic species complex

The phylogeny of *mmr-b* mirrors the species phylogeny based on *hsp82*, *coxI* and *its1* found here and in previous work [15-17]. The more rapid evolution of *mmr-b* relative to that of *hsp82* or *coxI* is a trait common to genes involved in reproduction such as gamete recognition and pheromone genes [31-34]*.* For *mmr-b*, this accelerated evolution is largely due to an increased rate in the fixation of non-synonymous mutations: *mmr-b* averaged double the dN and but half the dS of those of *coxI* and *hsp82*, resulting in an average dN/dS four to five times higher than for these “housekeeping” genes. Unlike *coxI* and *hsp82*, the accumulation of nonsynonymous differences in *mmr-b* shows no evidence of reaching a plateau with increasing synonymous difference, suggesting that functional constraints have not yet imposed the same limits on sequence divergence as has occurred with housekeeping genes. This pattern is apparent even when *hsp82* and *coxI* are only examined in the same dS range as *mmr-b*. An increased rate of non-synonymous substitution has been reported for several other sex-related genes [6,33,35]*.*

We predict that some of the observed amino acid changes cause differences in phosphorylation, glycosylation, and potentially the stability of the inferred helical secondary structure of the repeats. All of these changes could affect the ability of males to recognize MRP. We investigated whether the changes in the amino acid sequence of MRP repeats could be explained by positive selection. Though sliding window pairwise comparisons showed several regions where the average dN/dS exceeded 1, maximum likelihood analyses suggested that the observed divergence could be explained by neutral evolution, as none of the sites in the alignment or along tested branches have a dN/dS significantly greater than 1. Two branches have undergone a significant relaxation of selection, with an increase in both dN/dS and the proportion of codons under relaxed selection: the branch leading to Clade B Harvey and the branch leading to *B. ibericus*.

The tandemly arrayed organization of *mmr-b* repeat units, the variable number of repeats in different copies of the gene, the generally lower nucleotide divergence between repeats within a gene, and the near identity between gene copies within a genome and phylotype strongly suggest that *mmr-b* is homogenized by concerted evolution, likely involving gene conversion and/or unequal crossing over. This would also account for the lower observed accumulation of synonymous differences in *mmr-b* compared to *hsp82* and *coxI*. Concerted evolution has been implicated as the process maintaining the identity of repeats and producing copies with unequal numbers of repeats in reproduction-related genes in a wide range of organisms [31,36]. The lack of identity between full and terminal repeats of *mmr-b* is expected in concerted evolution, as unequal crossing over at the terminus of the repeat array is inhibited by the dissimilarity of flanking sequences [37]. As homologous genes have not been identified in distantly related taxa, it is unclear how to directly test whether the MPR gene family is evolving through a concerted or birth-and-death model, or a combination of both [27,38]. However, given the evidence presented above and absence of the large number of pseudogenes that are present in most other convincing cases of birth-and-death evolution of gene families, concerted evolution is currently the best-supported model of evolution of *mmr-b*[38].

Additional surveys at the population level will be necessary to determine the extent to which the many and variable versions of *mmr-b* found in each isolate are multiple gene copies rather than different alleles of a single gene, though the fact that we found copies with the same number of repeats in only five of the 27 isolates suggests that *mmr-b* exists as multiple loci in *Brachionus*. Also, the average nucleotide heterozygosity at the *hsp82* locus was 0.0016, compared to a much higher average nucleotide difference of 0.024 among *mmr-b* copies within an isolate, suggesting multiple copies homogenized by concerted evolution rather than allelic variation. Ongoing genome projects will eventually determine whether the copies of *mmr-b* are found on a single chromosome or are scattered throughout the genome, and will identify additional members of the MRP motif repeat gene family. Previously, we found two copies of *mmr-a* on a single fosmid from the *B. manjavacas* genome, separated by 7590 bp with no intervening open reading frame [27], suggesting that other members of the MMR family might also be found in tandem arrays.

While concerted evolution generally works to maintain identity between gene copies, it also means that when a mutation does occur, it may be propagated quickly through all of the repeats in all of the copies in the genome. For example, a single non-synonymous mutation, which would otherwise have minimal effect on the overall structure or function of the protein, could rapidly propagate across all repeats, resulting in a large-scale change in the properties of MRP that could lead to a decrease or loss of mate recognition between formerly compatible isolates. Such saltational change of a protein involved in mate choice could lead to rapid speciation with little morphological differentiation and a high degree of sympatry, as has occurred in the *B. plicatilis* cryptic species complex [39,40].

### Role of *mmr-b* in mate recognition and speciation

Despite the similarity in topology between the gene phylogenies for *mmr-b* (nuclear), *hsp82* (nuclear) and *coxI* (mitochondrial), both circling and copulation RI were only significantly correlated with amino acid distance of MMR-B. We cannot rule out the role of additional gene products on prezygotic reproductive isolation, but the decrease in mate recognition caused by knockdown of *mmr-b* expression by RNAi in *B. manjavacas* strongly suggests that mate recognition is controlled completely or at least primarily by a single protein on the surface of females and its corresponding receptor in males [28]. The positive correlation between RI (copulation only) and amino acid distance of HSP82, a gene product not expected to play a role in mate choice, is not unexpected given the similarity in the gene trees for *hsp82* and *mmr-b*. The correlation may indicate that only very small changes in MMR-B are needed to cause significant changes in mate recognition, or that the divergence in *hsp82* between species dates back to the time of speciation.

As shown by the positive correlation between *mmr-b* genetic distance and reproductive isolation, there was a gradient in mate recognition within the species complex. Complete reproductive isolation occurred only between the most distantly related phylotypes. We found evidence for nuclear introgression between more closely related phylotypes, indicative of interspecific reproduction. *Brachionus manjavacas* and a single strain of *B. plicatilis* s.s. shared in common individual repeats of *mmr-b*, but not the entire gene nor *hsp82* or mitochondrial *coxI* sequences. None of the other six strains of *B. plicatilis* s.s. had *mmr-b* repeats identical to those of *B. manjavacas.*

Within *B plicatilis* s.s., reciprocal crosses between isolates AUPEA006 and AUBUS001, with nearly identical MRP repeats, showed significantly lower rates of circling, but similar rates of copulation, in out-crosses relative to self-crosses. This suggests that the primary role of MRP is in recognition leading to copulation, and other proteins may play a significant role in promoting circling. Consistent with our finding of identical MRP repeats in Clade A Nevada and many *B. plicatilis* s.s. isolates, despite considerable nucleotide divergence in *mmr-b*, Suotoni *et al.* (2006) demonstrated that mate recognition between Clade A Nevada and *B. plicatilis* s.s. occurred at levels similar to self-crosses. Due to the unique MRP repeats shared by *B. plicatilis* s.s. isolates JPNAG062 and USGET006 but not other *B. plicatilis* s.s. isolates, we tested JPNAG062 in reciprocal crosses with AUBUS001, which has multiple copies of the dominant *B. plicatilis* s.s. MRP repeat. Circling occurred at rates similar to self-crosses, and males of JPNAG062 copulated with AUBUS001 females at a higher rate than with JPNAG062 females. In contrast, AUBUS001 males copulated with JPNAG062 females at a significantly lower rate, comparable to out-crosses between phylotypes. This suggests that the MRP repeats in the JPNAG062/USEGET006 group have diverged sufficiently to inhibit copulation, and that the corresponding male receptor in JPNAG062 has not yet evolved to recognize the new MRP phenotype. Despite the repeat shared by *B. manjavacas* and the *B. plicatilis* s.s. isolate BEARC010, *B. manjavacas* males did not copulate with BEARCO010 females at even the same rate as with females of other *B. plicatilis* s.s. isolates.

Previously, Suotoni *et al.* (2006) reported no mate recognition (circling + copulation) between the well-defined Clade B phylotypes Cayman and Almenara and a 4-fold reduction in mate recognition between Almenara and *B. ibericus*. We conducted reciprocal crosses between isolates of the Towerinniensis and the highly divergent Harvey phylotypes and found that males did not recognize females of the opposite phylotype. The extremely long branch leading to Clade B Harvey in all gene trees indicates rapid evolution of the entire genome for this isolate, perhaps indicative of a small population size leading to more rapid fixation of mutations or relaxed purifying selection. Our isolate of Harvey deviates from the rest of the species complex in other respects as well, including an apparent inability to thrive in large flasks (relative to culture tubes and well plates), extremely low production of males in mictic cultures, very low percentages of intraspecific circling and copulation in mating bioassays, and the retention of resting eggs internally in females. Harvey is the only isolate for which we were unable to hatch resting eggs in the laboratory.

## Conclusions

Attributing speciation directly to prezygotic reproductive isolation due to the evolution of *mmr-b* is supported by several lines of evidence in this study: *mmr-b* has a significantly higher nonsynonymous:synonymous nucleotide substitution ratio than other classes of genes, suggesting divergence resulting from sexual selection around the time of speciation; *B. plicatilis* is a species-rich group, as is expected in clades where sexual selection leads to speciation; there is partial premating isolation between populations of the same species; the complex is composed of closely related species that differ markedly in mating signals and preferences but that vary little in morphology or other traits; and there is asymmetry in mate preferences between males and females of different populations [6,41,42]. Dissecting the evolution of chemically mediated mate choice is often extremely difficult, as pheromone systems are frequently highly complicated, with multiple genes contributing to a complex blend of products constituting the species-specific chemical signal. Within the *B. plicatilis* complex, however, discrimination of compatible mates is determined by nearly identical proteins encoded by closely related copies of a single gene family, *mmr-b*[24,25,43].

Taken together, these finding suggest a model for mate recognition in the *B. plicatilis* species complex: A relatively simple set of extracellular globular coiled proteins encoded by *mmr-b* genes expose a variety of phosphorylated and glycosylated residues that serve as ligands to a receptor on the surface of males used to trigger copulation. These genes may have evolved specifically for mate recognition or, more likely, already existed as part of a family of extracellular matrix proteins with pleiotropic functions. Due to the nearly identical tandem-repeat nature of the *mmr-b* genes, concerted evolution is actively homogenizing repeats within a genome and altering the number of repeats in a transcript; as a result, single nucleotide changes proliferate across repeats in a genome. If these changes are nonsynonymous, an MRP protein can undergo a rapid change in overall ligand properties; this can affect the ability of males to recognize females carrying these converted alleles, erecting prezygotic barriers leading to insipient speciation. Because monogononts generally reproduce asexually and because copulation is not 100% dependent on MRP recognition, MRP repeats can drift to fixation in populations while compensatory changes in the male MRP receptor evolve. This process can result in neutral speciation without ecological drivers, and we see little or no evidence that positive selection is propelling variation in MRP. Indeed, there is a high degree of genetic diversity between *Brachionus* spp. populations on both local and global scales, despite a cosmopolitan distribution and potential for high gene flow; closely related but apparently reproductively isolated populations of *B. plicatilis* have been found in a number of aquatic ecosystems [22,39,44,45]. Future work could test the two predictions of this model: first, that rates of neutral sympatric speciation will be greater in permanent habitats where populations are not dependent on annual sexual cycles, and second, that while MRP may be evolving neutrally, changes in the male receptor will be under strong positive selection.

## Methods

### Culturing and DNA extraction

Rotifers were maintained in 25 ml culture tubes in 15 ppt artificial seawater (ASW, Instant Ocean). Rotifers were fed continuously and were supplied with fresh *Tetraselmis suecica* at approximately 4–6 x 10^5^ cells ml^-1^ weekly. The chlorophyte *T. suecica* was maintained in 2 L flasks in gently bubbled, 15 ppt ASW f/2 medium [46]. Both rotifer and algae cultures were kept at 21°C on a 12:12 h light:dark cycle. To collect and clean rotifers, cultures were sieved onto sterile 40 μm Nitex mesh and rinsed with 15 ppt artificial seawater to remove algae. Rotifers were rinsed with DI water (to stun the animals and prevent swimming that otherwise prohibits pelleting by centrifugation), pipetted into a 1.5 ml microcentrifuge tube, and pelleted by centrifugation. After removal of the supernatant by aspiration, genomic DNA was extracted using the DNeasy Blood and Tissue kit (Qiagen, Valencia, CA), according to the manufacturer’s instructions.

### Amplification and sequencing

Regions of three genes were amplified from rotifer DNA: a 711 bp region of the mitochondrial cytochrome oxidase gene, *cox1*[47], a 923 bp region of the nuclear 82 kD heat-shock protein gene, *hsp82*[48], and a region of variable length spanning the translation start and stop sites of MMR genes [25]. Primer sequences are listed in Table 6. Amplification reactions were performed in 25 μl volumes containing approximately 50 ng genomic DNA, 0.4 μM each primer, 200 μM dNTPs, 1 U TopTaq DNA polymerase (Qiagen, Valencia, CA), and 1X TopTaq PCR Buffer (Qiagen, Valencia, CA). PCR conditions were 4 min at 94°C, followed by 27 cycles of 30 s at 94°C, 20 s at T_a_, and 2 min at 72°C, followed by 7 min at 72°C, where T_a_ was 45°C – 55°C. The T_a_ was optimized for each gene and species combination.

**Table 6:** PCR Primers

**Gene**	**Primer name**	**Sequence (5′**-**3′)**
*mmr-a4*	MRPs3	ATGAAATCAATTTTATGTTCCTSCTG
	MRPurt3	TTAATCARAATAAAGAGGAAAAG
*mmr-a7*	MRPs3.1	ATGAAATCAATTTTATGTATCCTSCTG
	MRPutr1	GTATTTTTTATTTTTGATAAAAATCTG
*mmr-b*	2MRPs2	GTACCAGTYAAGCAAATAGCTGAACC
	2MRPutr1	ATATTTTAAATTAACCTGAACC
*hsp-82*	Hsp3:b	GARACNTTYGCNTTYCARGCN
	hsp8:b	RTGRTCYCCCARTCRTTNGT
*coxI*	HCO2198	TAAACTTCAGGGTGACCAAAAATCA
	LCO1490	GGTCAACAAATCATAAAGATATTGG

Amplification products were visualized on agarose gels, extracted using a MinElute Gel Extraction Kit (Qiagen), ligated into pCR4-TOPO vector, and transformed into *Escherichia coli* Top10 cells following the supplier’s protocol (Invitrogen, Carlsbad, CA). Plasmids were extracted from positive clones and sequenced in both directions using M13 forward and reverse primers with ABI Big Dye 3.1 chemistry on an ABI 3730xl Genetic Analyzer. The sequences were edited and assembled using Sequencher 4.10.1 (GeneCodes Corporation, Ann Arbor, MI).

Plasmids with *mmr* inserts too long to be fully sequenced by Sanger sequencing (approximately >1500 bp) were digested with *Eco*RI to isolate the insert. The insert was extracted from an agarose gel, purified with a MinElute column (Qiagen, Valencia, CA), and partially digested with *Nla*IV, which cuts once in each repeat (20 μl DNA, 0.4 U *Nla*IV, 1X NEB Buffer 4, 1X BSA, 32°C, 10 min). The digestion reaction was visualized on a 1% agarose gel, and bands between 800–1500 bp were excised and purified using a MinElute column, phosphatased using Shrimp Alkaline Phosphatase (USB, Clevland, OH, USA; 37°C for 60 min, 65°C for 20 min), and cloned into pCR4-TOPO as previously described. The insert sizes were checked by digestion with *Eco*RI, sequenced by Sanger sequencing, and assembled using Sequencher 4.10.1 using non-repetitive flanking sequences to guide alignment. Sequences were deposited in GenBank under accession nos. JX239140 - JX239171 (*cox1*), JX239172 - JX239200 (*hsp82*), and JX239201 - JX239258 (*mmr*); our analyses also included HM024709 (*B. manjavacas* RUS *cox1*) and AF143855 (*Brachionus calycifloris* FL *hsp82*).

### Phylogenetic analysis

Gene trees were generated using MrBayes 3.1 [49], with nucleotide frequencies and parameters for the GTR + gamma model (as selected by Modeltest 3.7 [50]) estimated independently for codon first + second positions and codon third positions. Two independent runs of four chains were run for 2 million generations and sampled every 100 generations; comparison of parameter estimates indicated convergence [51]. The first 1 million generations were discarded as burn-in and consensus trees examined with FigTree v1.2.2*.*

### Analysis for selection, structure, and post-translational modification

Measures of genetic distance and sliding window estimates of synonymous and nonsynonymous differences for *cox1*, *hsp82*, and *mmr-b* genes between pairs of isolates were determined using dnaSP [52] or MEGA 5.01 [53]. The codeml program within PAML [54] was used to search for evidence of positive selection throughout the species complex (codon frequencies F61 and MG3x4; nsites models 0, 1, 2, 7, and 8) and for differences in rates of evolution of MRP among clades [55]. We tested for significant differences in dN and dS using the Mann–Whitney *U*-test. The Garnier-Osguthorpe-Robson method (GOR V), Fragment Database Mining (FDM), Consensus Data Mining (CDM), and JPred3 were all used to predict the secondary structure of MRP proteins, based on translated DNA sequence ( http://gor.bb.iastate.edu/cdm/, [56-59]). NetPhos 3.1 was used to predict phosphorylation of MMR-B for all gene copies [60]. OGPet and ELM were employed to predict0 O-glycosylation and glucosaminoglycan-glycosylation, respectively (ogpet.utep.edu/OGPET/index.php, [61]).

### Mating Bioassays

Mating bioassays were performed to assess the degree of mate recognition within and between species following the procedure of Snell and Stelzer [26] with some modifications. Subcultures of isolates to be crossed were started by inoculating 100 gravid females into 200 ml of *T. suecica* culture of approximately 6 x 10^5^ cells ml^-1^ and were maintained as described above. Cultures were monitored for males daily. Once males were present, the cultures were sieved onto 100 μm Nitex mesh, which retained females and allowed the smaller males to pass through. Females were washed into a 15 ml centrifuge tube using 15 ppt sterile Instant Ocean water and vortexed for 30 seconds, causing them to drop their eggs. Amictic eggs were isolated into a 6 well dish and newly hatched females were collected after 1–2 hrs.

A complete bioassay was a full four-way cross (two self- and two out-crosses) with 10 replicates of 5-min exposures of 7 males to one young female for each cross. Six complete bioassays and four partial bioassays (because of the inability to produce enough males of some isolates) were conducted. For each replicate, males were added to a drop of water on a microscope slide printed with 10 6-mm diameter wells. Video recording (Sony Handycam) began immediately upon adding the female, and lasted 5 min. Encounters between males and females, circling of females by males, and copulations were recorded. We calculated average and standard error of the percent of circling resulting from encounters and of the percent of copulation resulting from circling. The Mann–Whitney *U*-test was used to determine the significance of differences in percent circling and percent copulation between the crosses. Prezygotic reproductive isolation (RI) was calculated as RI = 1 – (observed number of heterospecific matings/total matings) a variation on the method of Coyne and Orr [1] which allows for unequal numbers of crosses or where the two types of expected mating frequencies are unequal [62,63], using circling and copulation as separate measures of mating. The significance of correlations between RI and genetic distance was evaluated using the Pearson product–moment correlation coefficient, as the data were normally distributed.

## Competing interests

The authors declare that they have no competing interests.

## Authors’ contributions

KEG carried out the experiments; DBMW and KEG designed the experiments, analyzed and interpreted the data, and wrote the manuscript. Both authors read and approved the final manuscript.
